# Modulation of the Endocannabinoid System Following Central Nervous System Injury

**DOI:** 10.3390/ijms20020388

**Published:** 2019-01-17

**Authors:** Juan Zhou, Haneen Noori, Ian Burkovskiy, J. Daniel Lafreniere, Melanie E. M. Kelly, Christian Lehmann

**Affiliations:** 1 Department of Anesthesia, Pain Management and Perioperative Medicine, Dalhousie University, Halifax, NS B3H 4R2, Canada; chlehmann@dal.ca; 2 Department of Psychology and Neuroscience, Dalhousie University, Halifax, NS B3H 4R2, Canada; hn@Dal.Ca; 3 Department of Pharmacology, Dalhousie University, Halifax, NS B3H 4R2, Canada; ian.b@Dal.Ca (I.B.); lafreniere@Dal.Ca (J.D.L.); memkelly@icloud.com (M.E.M.K.); 4 Department of Microbiology and Immunology, Dalhousie University, Halifax, NS B3H 4R2, Canada; 5 Department of Physiology and Biophysics, Dalhousie University, Halifax, NS B3H 4R2, Canada

**Keywords:** central nervous system injury, endocannabinoid system, immunodepression

## Abstract

Central nervous system (CNS) injury, such as stroke or trauma, is known to increase susceptibility to various infections that adversely affect patient outcomes (CNS injury-induced immunodepression—CIDS). The endocannabinoid system (ECS) has been shown to have immunoregulatory properties. Therefore, the ECS might represent a druggable target to overcome CIDS. Evidence suggests that cannabinoid type 2 receptor (CB_2_R) activation can be protective during the early pro-inflammatory phase after CNS injury, as it limits neuro-inflammation and, therefore, attenuates CIDS severity. In the later phase post CNS injury, CB_2_R inhibition is suggested as a promising pharmacologic strategy to restore immune function in order to prevent infection.

## 1. Clinical Background and Pathophysiology

Central nervous system (CNS) injury affects millions of patients annually and is one of the most common causes of mortality or disability globally [1]. Central nervous system injury includes pathologies such as stroke, traumatic brain injury and spinal cord injury. Stroke is the second leading cause of death worldwide and is an acute CNS injury resulting from obstructed blood supply to the brain or bleeding into the brain [2]. In the United States, hemorrhagic stroke accounts for 13% of all strokes, with those due to ischemia representing 87% [3]. The pathophysiology of stroke centers on the delicate nature of brain tissue, in which short periods of ischemia and/or glucose deprivation can trigger cellular death (via necrosis and apoptosis), leading to tissue loss [4]. The tissue at, and downstream from, an occluded and/or damaged vessel is most starved of oxygen/glucose, as compared to nearby tissue which may receive some diffusion from other neighbouring vasculature. The area of infarct (necrotic tissue) is, thus, generally surrounded by an area of tissue where recovery may be possible following reperfusion, referred to as the “penumbra”. Treatment for acute ischemic stroke (AIS) may involve arterial thrombectomy or thrombolysis (via recombinant tissue plasminogen activator—rtPA), which can generally be safely initiated within a few hours post-AIS [5]. Treatment for hemorrhagic stroke aims to decrease bleeding and reduce increased intracranial pressure, with potential interventions including anti-hypertensives, mannitol, reversal of coagulopathy, ventriculostomy and/or craniotomy [6,7,8]. A number of pathologic changes occur at the level of the neuron during ischemia, which lead to both necrotic and apoptotic processes, including ATP depletion, the accumulation of reactive oxygen species, cellular edema, acidosis, and changes in ionic concentrations [4]. 

A range of post-stroke complications contributes to mortality and morbidity. In the first week following stroke, cerebrovascular disease is the most common cause of death, followed by pulmonary embolism (weeks 2–4) and bronchopneumonia (months 2 and 3) [9]. Infections are common in post-stroke patients, with an incidence of pneumonia at 22% and urinary tract infection (UTI) at 24% [10]. Evidence suggests an intricate link between stroke pathophysiology and systemic immune function [11,12,13,14,15,16], where an initial hyper-inflammatory phase may be followed by a period of systemic immunosuppression (CNS injury-induced immunodepression—CIDS) [17]. The later phases of stroke include the subacute phase, which lasts hours to days after onset, and the chronic phase, which lasts days to months and can continue for the remainder of the patient’s life [18]. The incidence of fatal infections is linked to severity of CNS injury and the status of the immune system [19,20]. Accordingly, the potential for post-stroke antimicrobial prophylaxis has been assessed and showed conflicting results [21]. Given these findings, the potential for more complex approaches to prevent CIDS-related infections, namely, immunomodulation, are both novel and promising. 

## 2. CNS Injury-Associated Immune Response

**Figure 1:**
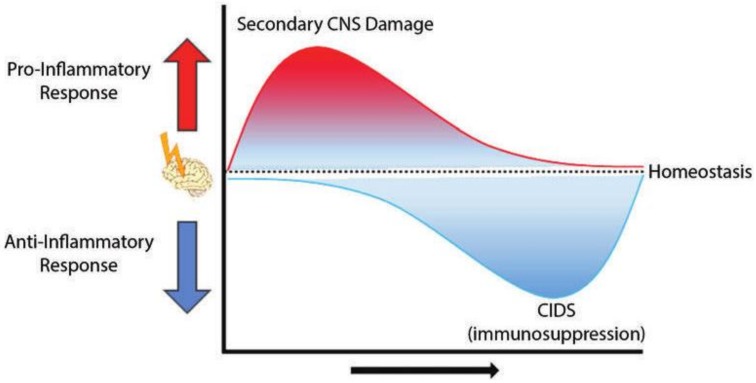
Graphic representation of the dynamic changes in immune response after CNS injury. The initial trigger (e.g., traumatic brain injury, stroke, etc.) causes a strong pro-inflammatory response—influx of immune cells, activation of resident microglia, production of inflammatory cytokines and other pathophysiologic changes. Combined with the dysfunction of the blood–brain barrier (BBB), the strong inflammatory response causes secondary CNS damage and exacerbates the injury size. As a compensatory and neuroprotective mechanism, anti-inflammatory pathways are activated in the brain, resulting in an immunosuppressed state which also affects the peripheral immune response (CNS injury-induced immunodepression—CIDS). Figure used with permission from the publisher in reference [24].

After acute CNS injury, a sequence of events occurs at the primary site due to deprivation of oxygen and nutrients. These include tissue hypoxia, which causes rapid neuronal death and the release of various danger-associated molecular patterns (DAMPs) that activate microglia (brain resident immune cells) and astrocytes; activated cells produce large amounts of inflammatory cytokines, chemokines and reactive oxygen species which subsequently activate endothelial cells and disrupt blood-brain barrier (BBB) integrity, resulting in recruitment of systemic immune cells that infiltrate the infarct site. Neutrophils, macrophages, and mast cells, as components of innate immunity, are early infiltrates that produce pro-inflammatory cytokines, proteolytic enzymes and neurotoxic mediators, which cause further breakdown of endothelial tight junctions and aggravate BBB injury [22]. The initial infarct area can progress over time when more resident immune cells and endothelial cells are activated, leading to more recruitment of peripheral immune cells into the brain and further damage of BBB. Collectively, these events facilitate a massive “second wave” of immune cell entry into the brain parenchyma, causing significant secondary cell death outside the original injury area and exacerbating the pathology of CNS injury, i.e., neuroinflammation [23,24] (Figure 1). The level of neuroinflammation is highly dependent on the severity, duration, and the anatomical context of the CNS injury.

Adaptive immune responses also play a critical role in CNS injury. Natural killer (NK) cells, T cells and B cells were detected in experimental cerebral ischemic brain as early as 3 hours following acute stroke [25]. Transgenic mice deficient in CD4^+^, CD8^+^ or γδ T cells showed decreased infarct volume and improved neurological function, whereas restoring the lymphocyte population in these immunodeficient mice by adoptive transfer of wild-type splenocytes reversed the protective effect, suggesting a detrimental role of such lymphocyte population in brain injury [26,27]. It has been suggested that NK and CD4^+^ T cell-caused neurotoxicity is mediated by secretion of IL-17 and INF-γ, whereas CD8^+^ T cells generate direct neuronal cytotoxicity [28]. On the other hand, regulatory T (Treg) and B cells are generally characterized as anti-inflammatory and disease-limiting protective cells [22]. However, evidence also showed that reduction of infarct size was observed in Treg-deficient mice [29], and B cell deficient mice failed to show reduction of neurological deficits in ischemic stroke [26]. This may be interpreted by various factors, such as the experimental model used or evaluation time point of stroke.

To prevent the excessive action of pro-inflammatory cytokines after the initial beneficial effects, the immune system releases several anti-inflammatory mediators, such as IL-10, IL-1 receptor antagonist and soluble tumor necrosis factor (TNF) receptors, to generate a cascade of compensatory anti-inflammatory responses. Meanwhile, an initial acute CNS injury also activates immune inhibitory pathways, generating a systemic brain-mediated immunosuppression, i.e., CIDS, to minimize secondary damage to healthy CNS tissue (Figure 1) [17,24]. In addition, CIDS is also considered to provide protection from autoimmunity by suppression of autoreactive lymphocytes [16]. Due to the dysfunction of BBB, immune cells get access to the CNS and encounter the antigens that are normally sequestered in the brain and are invisible to the immune system. It was shown that autoreactive lymphocytes to CNS antigens were present in the spleens and lymph nodes in experimental stroke and stroke patients, and the impact of autoimmune response on stroke outcome was dependent on the autoantigen epitopes [30,31]. Although the overall consequences of CIDS are unclear and differ between patients, CIDS is believed to be the main reason for increased susceptibility to infections, a leading cause of death in patients with acute CNS injury due to impaired immune function [32]. Further, CIDS is known to compromise the immune response to infections such as pneumonia and urinary tract infection, worsening stroke outcome. The severity of immunosuppression is dependent on the initial size of CNS injury and the underlying medical conditions of the patient.

The endocannabinoid system (ECS) is a ubiquitous system with established roles in the modulation of a range of physiological and disease processes. In particular, the link between the ECS and immune function qualifies as a promising target for immunomodulation. 

## 3. The Endocannabinoid System in CNS Injury

### The Endocannabinoid System

**Figure 2:**
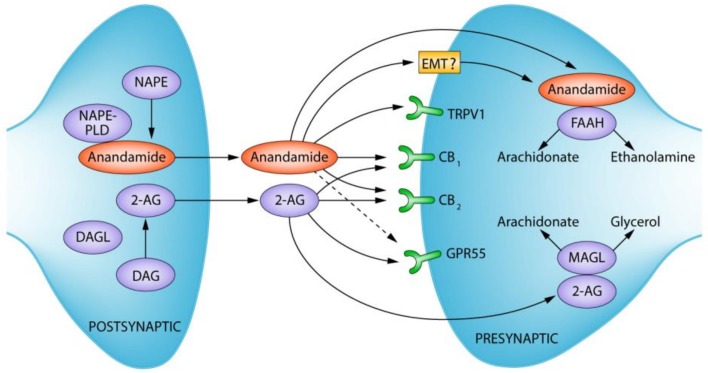
Overview of the endocannabinoid system (ECS), outlining key endocannabinoids, cannabinoid receptors, and related enzymes involved in endocannabinoid synthesis and degradation. DAG: Diacylglycerol; DAGL: Diacylglycerol lipase; 2-AG: 2-arachidonoylglycerol; NAPE: *N*-acyl-phosphatidylethanolamine; NAPE-PLD: *N*-acyl-phosphatidylethanolamine-specific phospholipase D; TRPV1: Transient receptor potential cation channel subfamily V member 1; CB1: Cannabinoid receptor 1; CB2: Cannabinoid receptor 2; GPR55: G protein-coupled receptor 55; MAGL: Monoacylglycerol lipase; FAAH: Fatty acid amide hydrolase; EMT: Endocannabinoid membrane transporter—“?” denotes controversy surrounding the presence of said transporter. Figure used with permission from the publisher in reference [47] (version used modified by reference [48]).

The discovery of Δ^9^-tetrahydrocannabinol (THC) by Gaoni and Mechoulam in 1964 was the first milestone in a series of scientific discoveries that resulted in the description of the ECS [33]. The ECS is composed of the cannabinoid receptors, their endogenous lipid-based ligands and cognate synthetic and degradative enzymes [34]. This ubiquitous system has been shown to have a key role in a range of physiological and disease processes [33,34,35,36,37,38,39]. The two well-identified cannabinoid receptors include the cannabinoid type 1 receptor (CB_1_R) and the cannabinoid type 2 receptor (CB_2_R), both G-protein coupled receptors linked to G_i/o_ [40]. The CB_1_R is highly expressed throughout the CNS, as well as other peripheral tissues, while CB_2_R is expressed primarily in non-neuronal tissues, particularly on immune cells such as phagocytes, B-cells, T-cells and natural killer (NK) cells [41,42,43]. The levels of CB_2_R mRNA displays major variation in human blood leukocytes with a rank order of B lymphocytes > NK cells > monocytes > polymorphonuclear neutrophils > CD8^+^ lymphocytes > CD4^+^ lymphocytes [44]. The endogenous cannabinoids, termed endocannabinoids, include anandamide (*N*-arachidonoylethanolamine or AEA) and 2-arachidonoylglycerol (2-AG). These endocannabinoids are generated from membrane lipids in an “on-demand” fashion, via the actions of both synthetic and degradative enzymes [45,46]. The main enzyme that is involved in AEA biosynthesis is *N*-acylphosphatidylethanolamine-phospholipase D (NAPE-PLD), which cleaves *N*-arachidonylphosphatidylethanolamine (*N*-ArPE) into AEA and phosphatidic acid [45]. The 2-AG biosynthesis begins with phospholipase C-mediated hydrolysis of membrane phospholipids, yielding diacylglycerol (DAG) that is then converted to 2-AG by diacylglycerol lipase (DGL). The 2-AG is then inactivated by monoacylglycerol lipase (MAGL), while AEA is hydrolyzed by FAAH [45,47] (Figure 2).

The ECS plays an important role in the regulation of the immune system in response to inflammation. A growing body of evidence shows an upregulation of the ECS during both local and systemic inflammation [49]. Increased CB_2_R expression is reported on neutrophils, macrophages, and lymphocytes, and activation of CB_2_R is associated with anti-inflammatory effects including reduced macrophage and neutrophil numbers at the site of infection and decreased pro-inflammatory cytokine production [50,51].

In CNS injury, significant increased CBR expression and endocannabinoid levels were found [52,53,54]. Activation of CB_1_R-related pathways has been reported to decrease CNS excitability and cell death by controlling glutamate homeostasis and reducing glutamate toxicity, whereas activation of CB_2_R on cerebral immune cells limits post-ischemic neuroinflammation [55]. Increased CB_2_R but decreased CB_1_R mRNA expression was found in the ischemic rat cortex which was associated with increased inflammatory mediator levels, such as IBA1 and TLR4 [52,56]. However, the ECS involvement in CNS injury is complex and the experimental data are controversial.

Microglia are resident CNS cells that play a critical role not only in immune defense but also in tissue repair and neuronal homeostasis. Microglia cells possess phenotypical and functional features related to macrophages and are activated early after stroke. They can be activated by free radicals, other DAMPs and high-mobility group box 1 (HMGB1) protein, and undergo maturation, differentiation and activation processes [22,57]. Upon activation, these cells produce various cytokines, including TNFα, IL-1 and IL-6, express complement receptor, CD11/CD18 complex, and major histocompatibility complex (MHC) class I and II antigens, which are key components of antigen presentation in activation of the adaptive immune response [57]. Activated microglia are phagocytic, able to process antigens and exert cytolytic functions. Their cytokine production ranges from pro-inflammatory to anti-inflammatory profiles depending on their activation state. In resting microglia cells no CB_2_R mRNA was measurable; however, upon activation microglia cells upregulate CB_2_R mRNA [58,59]. Evidence suggests that microglia cells are essential in reduction of stroke-related damage, because microglia-depleted mice showed larger infarct volume than non-depleted mice [60]. In addition, 2-AG triggered migration of microglia is associated with CB_2_R expression [61]. Taken together, there is growing evidence suggesting that CB_2_R-mediated immune response in the CNS may be exerted in large part through microglia. 

Modulation of CB_2_R signaling has shown neuroprotection and reduction of neuro-inflammation in several experimental models. The use of CB_2_R agonists in experimental middle cerebral artery occlusion (MCAO) reduced leukocyte infiltration into the ischemic site of the brain, decreased neuroinflammation and improved neurological functional outcome [56,62,63,64]. In addition, activation of the CB_2_R in traumatic brain injury [65] and spinal cord injuries [66] dampened neuroinflammation and reduced neurological impairment. The neuroprotective effect of CB_2_R agonists was diminished in CB_2_R knockout mice, confirming that a protective mechanism through increased CB_2_R signaling is accountable for reduction of CNS injury induced inflammation [67]. 

## 4. Early Post-Stroke Phase

### 4.1. Overview of Pathophysiology

An important task of the inflammatory response is the neurological repair process following an acute stroke. This process, as described earlier, is initiated by the release of pro-inflammatory mediators followed by recruitment of leukocytes to the CNS [14,18]. Disruption of the BBB disturbs the homeostatic microenvironment of the CNS and allows the extravasation of blood components into the brain and compromises normal neuronal function [68].

Early post-stroke inflammation may act as a double-edged sword [32,69]. In terms of benefits, the inflammatory process plays an important role in clearing damaged tissue, in addition to a role in tissue remodeling and regeneration [32]. Specific inflammatory mediators possess neuroprotective and neuro-regenerative properties in the post-stroke setting [32]. However, depending on the initial extent of CNS damage, stroke-induced inflammation can also be detrimental and contribute to secondary pathologies [69]. Inhibition of this initial inflammatory response, therefore, is a viable therapeutic strategy and has been at the forefront of recent studies and investigations. 

### 4.2. Potential for ECS Modulation 

Modulation of the ECS, namely via activation of CB_2_R during the early post-stroke phase, may hold therapeutic promise. A number of experimental studies have investigated the role of cannabinoid receptors in ameliorating CNS injury and immune responses. Most of the studies demonstrated anti-inflammation and neuroprotection effects upon CB_2_R-activation [52,56,63,70]. Following CNS injury, CB_2_R expression is upregulated on both resident CNS and infiltrating immune cells that originate in the periphery [56,70,71]. In a MCAO rat model, CB_2_R mRNA expression in the ischemic cortex was elevated over 20 fold on day 2 and peaked over 40 fold on day 5 [52]. Pre-MCAO treatment with CB_2_R agonist, AM1241, significantly reduced glutamate-mediated neurodegeneration in primary cortical neurons in cultures and reduced brain infarct volume and neurological Bederson scores [52].

Using different selective CB_2_R agonists (O-3853, O-1966) in a transient MCAO-induced cerebral ischemia model in mice, Zhang et al. showed a decreased immune response and neuroprotective effect upon CB_2_R activation [62]. They found that treatment with a CB_2_R agonist 1 h prior to induction of ischemia significantly decreased leukocyte rolling and adhesion in cerebral venules. The treatment also reduced infarct volumes and improved motor function evaluated at 24 h post-ischemia [62]. The diminished leukocyte–endothelial interaction by CB_2_R agonist treatment indicates that reduced post-ischemia inflammatory response may contribute to the neuroprotective role of CB_2_R activation. Additionally, the neuroprotective effect of CB_2_R was confirmed in CB_2_R knockout mice [63].

Although pre-treatment with CB_2_R agonist has significant neuroprotective effects in CNS injury, it is not feasible in clinical settings. Therefore, administration of a CB_2_R agonist after onset of ischemic injury was performed by Zarruk and coworkers using a permanent MCAO-induced ischemia model in mice. They demonstrated that treatment with the selective CB_2_R agonist JWH-133 10 min after occlusion significantly reduced microglial activation, decreased inflammatory gene expression, reduced brain infarct size and ameliorated neurological impairment [56]. This effect was absent in CB_2_R knockout mice and reversed with administration of a CB_2_R antagonist (SR144528) [56]. In their experimental model, MCAO increased CB_2_R mRNA but decreased CB_1_R mRNA expression. These data suggest that CB_2_R activation plays a critical role in the protection of stroke-induced brain damage. The protective effect was due to inhibition of microglia/macrophage activation and an anti-inflammatory mechanism mediated by CB_2_R [56].

Using genetic knockout of CB_2_R mice, Amenta et al. showed exacerbated expression of pro-inflammatory mediators, such as TNF-α and intercellular adhesion molecule 1 (ICAM-1) in the mice after traumatic brain injury. In addition, treatment with CB_2_R agonists (0-1966, JWH-133) in wildtype mice attenuated TNF-α and ICAM-1 levels, decreased inducible nitric oxide synthase (iNOS) mRNA expression, and reduced BBB permeability in the injured cortex. Immunohistochemistry confirmed that iNOS was expressed by macrophage and microglia in the injured cortex. These data confirm the immunosuppressive effects of CB_2_R activation in CNS injury [70]. 

In addition to synthetic CB_2_R agonists, effects of the phytocannabinoid, cannabidiol (CBD), on neuroprotection has also been studied [72,73,74,75]. Cannabidiol is considered to act as a negative allosteric modulator at CB_1_R, a partial agonist at CB_2_R and an agonist at serotonin 5HT1A receptors [74,76,77,78]. Using a 30-min hypoxic-ischemic (HI) brain injury model in newborn pigs, Pazos and coworkers demonstrated that administration of CBD 30 min after HI induction significantly reduced neurexcitotoxicity, oxidative stress and neuroinflammation, suggesting a neuroprotective effect of CBD [73]. This CBD-mediated neuroprotection was reversed by co-administration of CB_2_R antagonist, AM630, or 5-HT(1A) antagonist, WAY100635 [73]. It was suggested that this CBD-mediated neuroprotection might act through indirect action of CB_2_R, such as through heterodimers with 5HT1A receptors [73].

The role of CB_1_R activation in neuroprotection is controversial. An initial study with CB_1_R knockout mice demonstrated increased severity of stroke, including increased mortality, infarct size, and neurological deficits, with decreased blood flow in the infarct penumbra following cerebral ischemia reperfusion in CB_1_R^−/−^ animals [79]. Then, Caltana and co-workers demonstrated neuroprotective properties of CB_1_R agonist, arachidonoyl-2′-chrooroethyamide (ACEA), in MCAO-induced brain injuries in mice. They showed reduced deterioration in motor activity and neuronal death as well as reduced astrocytic and microglial activation in the mice with brain injury and ACEA treatment, suggesting a neuroprotective role of CB_1_R activation [80]. However, other studies demonstrated that selective antagonists of CB_1_R have a neuroprotective role [53,81]. Interestingly, recent evidence showed a surprising finding that double knockout of CB_1_ and CB_2_ receptors showed improved outcomes [82]. The role of CB_1_R in neuroinflammation and protection needs to be futher investigated.

## 5. Late Post-Stroke Phase

### 5.1. Overview of Pathophysiology

The later phases of stroke include the subacute phase, which lasts hours to days after onset, and the chronic phase, which lasts days to months and can continue for the remainder of the patient’s life [18]. Central nervous system injury can lead to secondary immunodeficiency, i.e., CIDS [17]. This (CIDS) is likely a result of a compensatory response to CNS inflammation [83], where local immunosuppressive mechanisms are not CNS-restricted and lead to the development of systemic immunosuppression [84]. Both experimental and clinical lines of evidence suggest that the mechanism by which CIDS manifests is through activation of the sympathetic nervous system and the hypothalamic–pituitary axis [85]. Although it is not completely known at present which signals stimulate these systems to downregulate the immune response after stroke, pro-inflammatory cytokines are most likely the triggers of the process [32]. Although inflammatory response after stroke initially promotes healing and eliminates necrotic cells, excessive inflammation can induce CIDS and may increase incidence of secondary infections. Prevention of CIDS onset and development is an important therapeutic target to improve the outcome of patients after CNS injury. 

Since the immune response plays an active role in the pathology and prognosis of CNS injury, it is important to understand the time course of events that lead to inflammation in the ischemic brain. Experimentally, brain injury causes a time-dependent recruitment and activation of many inflammatory cells such as monocytes/macrophages, neutrophils, and T-cells [86]. During the subacute phase, leukocytes will release cytokines and chemokines which will further fuel the inflammatory response in the brain, including BBB damage, neuronal death, brain edema and hemorrhagic transformation [18]. Studies also suggest that lymphocytes are recruited into the brain during the later stages of stroke, and many subtypes of these lymphocytes have been involved in the late consequences of ischemic stroke [18]. 

### 5.2. Potential ECS Modulation During Late Post-Stroke Phase

Recent evidence has shown that ECS modulation may be useful in the late-phase to reduce the severity of CIDS [87]. As previously mentioned, CB_2_R is upregulated following stroke, which may subsequently contribute to the immunodepression of CIDS [88]. Although activating CB_2_R during the early post-stroke phase can be neuroprotective in terms of reducing the CNS inflammatory response, during the late post-stroke phase, when the patient is immunocompromised, it follows that inhibition of CB_2_R activity may be beneficial in order to improve peripheral immune response [87]. Using a mouse model of CNS injury generated by left common carotid artery ligation followed by exposure to hypoxia, we demonstrated a reduced systemic immune response to LPS 24 hours after induction of stroke [87]. Treatment of the immunocompromised mice with a CB_2_R antagonist significantly improved the immune response to endotoxin challenge. This treatment did not change the infarct volume and severity of neurological dysfunction [87]. The study suggested that the ECS is indeed involved in the impaired immune function following stroke and that ECS modulation, particularly CB_2_R inhibition, may be useful in treating CIDS. 

Although preclinical data indicates that CB_2_R activation is neuroprotective when a CB_2_R agonist is administered at the early phase of CNS injury, as described above, studies also demonstrated that the protective effect may be diminished if CB_2_R agonist is administrated at the late phase after stroke. Yu et al. showed that pre-MCAO treatment with the CB_2_R agonist, AM1241, decreased brain inflammation and infarct size and improved neurological behavior, but that treatment at 2 days post-MCAO only reduced the number of microglia (IBA1^+^), CD4^+^ and CD8^+^ cells in the peri-lesioned cortex, with no significant reduction in the infarct volume and neurological scores evaluated on day 6 post-MCAO [52]. In another study, the CB_2_R selective agonist, GW405833, administered 30 min post brain damage induced by hypoxia-ischemia in rats did not show neuroprotection 15 days post brain injury [89].

Taken together, present data suggest that many factors are able to influence the efficacy of CB_2_R modulation in CNS injury, including the time, route of administration and dose of the specific drug.

## 6. Conclusions and Future Directions

Available evidence indicates that the ECS is intricately involved in the immune consequences following CNS injury. Selective modulation of CB_2_R has been shown to hold therapeutic potential in the post-stroke setting. Depending on the initial extent of CNS damage, stroke-induced inflammation can have direct harmful effects on the peripheral immune response (i.e., CIDS), in addition to contributing to the development of secondary CNS pathologies. Attenuation of initial inflammation via CB_2_R activation is therefore a viable therapeutic strategy for stroke patients. Due to its anti-inflammatory properties, CB_2_R activation can be protective during the early pro-inflammatory phase of stroke as it initiates immunosuppressive mechanisms that limit neuro-inflammation. A number of studies have investigated the role of CB_2_R activation in cerebral ischemia models and confirmed a neuroprotective effect of CB_2_R activation. Although this strategy is beneficial during the early phase of stroke, long-term CB_2_R activation would likely contribute to the development of CIDS, worsening neurological outcomes and increasing mortality. Therefore, recent evidence suggests that inhibiting CB_2_R activity at late phase can reduce post-stroke immunodepression and limit the consequences of CIDS. Although results indicate therapeutic potential with this strategy, stimulating the immune system could theoretically lead to increased neuro-inflammation. For this reason, future studies are needed to better characterize the effects of ECS modulation in this setting, as well as to define an appropriate therapeutic window and parameters for treatment. Biomarkers of immune status and CNS-injury are likely key in assessing progression to CIDS and tailoring immunomodulatory strategies.
